# Human airway material characterization via inverse finite element analysis and neural network surrogate

**DOI:** 10.1007/s10237-026-02081-7

**Published:** 2026-06-03

**Authors:** Arif Badrou, Crystal A. Mariano, Talyah M. Nelson, Mona Eskandari

**Affiliations:** 1 Department of Mechanical Engineering, University of California Riverside, Riverside, CA USA; 2 BREATHE Center, School of Medicine, University of California Riverside, Riverside, CA USA

**Keywords:** Human airway, Inverse finite element analysis, Central airway obstruction, Lung cancer, Anisotropy, Biaxial tensile testing

## Abstract

Central airway obstruction can be caused by lung cancer and may severely diminish respiratory function to necessitate airway stenting. However, the mechanical properties of airway tissues remain poorly characterized, leading to a mismatch between stent mechanical behavior and airway compliance, which can reduce stent biocompatibility and clinical effectiveness. Such mismatches can result in abnormal stress transfer at the stent–airway interface and cause stent migration, local tissue irritation or inflammation, and impaired long-term performance. Predictive models are often used in treatment planning, however, without a comprehensive understanding of airway properties, advancements in modeling remain highly limited. In this study, we develop an experimental to numerical pipeline for identifying the mechanical properties of the human airway branching network, specifically the trachea, right bronchus, and left bronchus tissues. Biaxial planar tensile tests are used to capture the tissues’ unique, anisotropic mechanical response in order to inform the computational routine—where inverse finite element analysis is employed to calibrate a Holzapfel–Gasser–Ogden constitutive model. To overcome the computational burden of traditional IFEA, a neural network (NN) surrogate model is trained to enable rapid material identification. We identify parameter values for the various tissues, such as *C*_10_ = 0.53 ± 0.25 kPa, *k*_1_ = 0.17 ± 0.30 kPa, *k*_2_ = 6.1 ± 2.0, and *κ* = 0.08 ± 0.01 for the trachea. Notably, this NN-approach reduced computational time from weeks to mere minutes, enabling fast material characterization. Thus, we also provide an efficient modeling framework and user-friendly MATLAB application for future studies. Ultimately, the findings here critically contribute to our current understanding of airway biomechanics and provide essential data for accurate modeling and optimization of airway stenting strategies.

**Supplementary Information:**

The online version contains supplementary material available at 10.1007/s10237-026-02081-7.

## Introduction

Lung cancer is the leading cause of cancer-related mortality worldwide, with more than two million new cases diagnosed in 2018 (Chaitanya Thandra et al. 2021). By 2030, lung cancer is projected to account for 3% of total global deaths (Yousefi et al. 2023). Beyond its devastating human toll, lung cancer also imposes a significant and irreversible economic burden. In the USA alone, the total economic impact of lung cancer including medical costs, lost productivity, and premature mortality exceeded $13 billion in 2019 (Kratzer et al. 2024). Among those affected, approximately 30% of patients develop central airway obstruction (CAO), primarily involving the trachea, right primary bronchus, and left primary bronchus (Saji et al. 2010). Without timely intervention, CAO drastically reduces survival rates (Umar et al. 2023). As such, it is of utmost significance to enable solutions to mitigate CAO.

Airway stenting is one of the most effective interventions for CAO—where the insertion of a hollow tube restores airflow to improve lung oxygenation (Salguero et al. 2024; Saji et al. 2010). Despite recent advancements, such as more effective patient-specific stent designs, complications still prevail (Li et al. 2023). For instance, long-term issues such as stent migration (i.e., position shift) are common: Dialani et al. (Dialani et al. 2008) reported how stent migration away from surgical placement in the proximal right bronchus led to partial collapse of a patient’s right upper lung lobe. Such complications occur frequently, in 5–17% of CAO patients, and are often attributable to design and implementation issues, highlighting the need for improved stent structures and placement strategies (Madan et al. 2024).

Numerical models play a crucial role in minimizing complications such as stent migration, as they provide preoperative insights into the mechanical response of stented airways (Malvè et al. 2011; Saeidi et al. 2023). However, the accuracy of these models fundamentally depends on precise characterization of airway tissue mechanical properties. Current finite element (FE) models of airway stenting suffer from a critical limitation: they predominantly assume isotropic material behavior (Farahani et al. 2023), despite recent experimental evidence demonstrating the highly anisotropic nature of mammalian airways (Eskandari et al. 2018; Sattari et al. 2023). This represents a current limitation that can compromise the predictive capability of stent simulations.

Previous studies have characterized the mechanical properties of the trachea across different species, including humans, using a range of experimental approaches, most commonly uniaxial tensile testing or compression (Trabelsi et al. 2010; Safshekan et al. 2016; Huang et al. 2021; Jau-Yi Wang et al. 2011). While these studies have provided valuable baseline insights into airway mechanics, simplified loading conditions such as uniaxial tension are known to offer a limited representation of the anisotropic and nonlinear behavior of soft tissues, motivating the need for more comprehensive experimental and modeling frameworks (Jiang et al. 2021). As a result, accurately translating such experimental measurements into constitutive material parameters suitable for computational modeling remains a nontrivial challenge.

The two common approaches for identifying material properties in numerical models are direct stress–strain curve-based parameter fitting and inverse finite element analysis (IFEA). Direct curve-based fitting involves optimizing parameters of a function that describes the material behavior, minimizing the difference between experimental and numerical data. Although this approach is relatively straightforward, it provides only an approximate estimate of tissue properties because it relies on a predefined mathematical model that may not fully capture the complexity of the tissue's mechanical response, particularly in fitting the global response of the data (i.e., how the force measure responds to an imposed displacement). The second approach, IFEA, uses an FE model to simulate the experimental setup and test results (e.g., tissue biaxial testing), where the material parameters are adjusted until the simulation results agree with the experimental data (Bäker and Shrot 2013). Consequently, IFEA is known for its precision, as the FE model incorporates the actual physics and boundary conditions of the soft tissues, which are often too complex to describe with a simplified curve-fitting mathematical function (Kauer et al. 2002). However, IFEA’s complexity can make the problem ill-posed and is computationally expensive. In particular, for nonlinear constitutive models with multiple parameters, different combinations of material parameters can produce very similar force–displacement responses, leading to non-unique solutions. In addition, strong coupling between parameters and sensitivity to experimental noise and boundary conditions further complicate parameter identification, requiring repeated simulations during optimization (Bäker and Shrot 2013; Liu et al. 2014). For example, Chawla et al. (Chawla et al. 2009) reports that characterizing human passive muscle using IFEA requires approximately 10 h of computational time to obtain optimized material parameters.

Despite these methodological advances, a critical gap remains: Existing studies have not yet systematically combined biaxial experimental characterization of human airway tissues with a computationally efficient inverse finite element framework capable of identifying anisotropic material parameters in a manner suitable for airway stenting applications. In this study, we address this gap by introducing a new approach to airway tissue characterization using IFEA, establishing an integrated experimental-to-modeling pipeline aimed at improving physiological relevance while reducing computational cost. To this end, we present the first biaxial characterization of the mechanical properties of human airway tissues, including the trachea, right bronchus, and left bronchus tissue samples, with methods based on recent advancements from our group (Mariano and Eskandari 2024; Sattari et al. 2023; Mariano et al. 2023; Nelson et al. 2024). These data are then used in a numerical pipeline that efficiently identifies material parameters using an inverse approach. By integrating a feedforward neural network (NN) as a surrogate model within the optimization loop, our method drastically reduces the computational cost of FE simulations, enabling rapid and accurate calibration. This new application not only enhances current models of human airways but also provides a highly efficient framework for future research, significantly improving simulations related to soft tissue characterization.

## Materials and methods

### Experimental to numerical framework

**Fig. 1:**
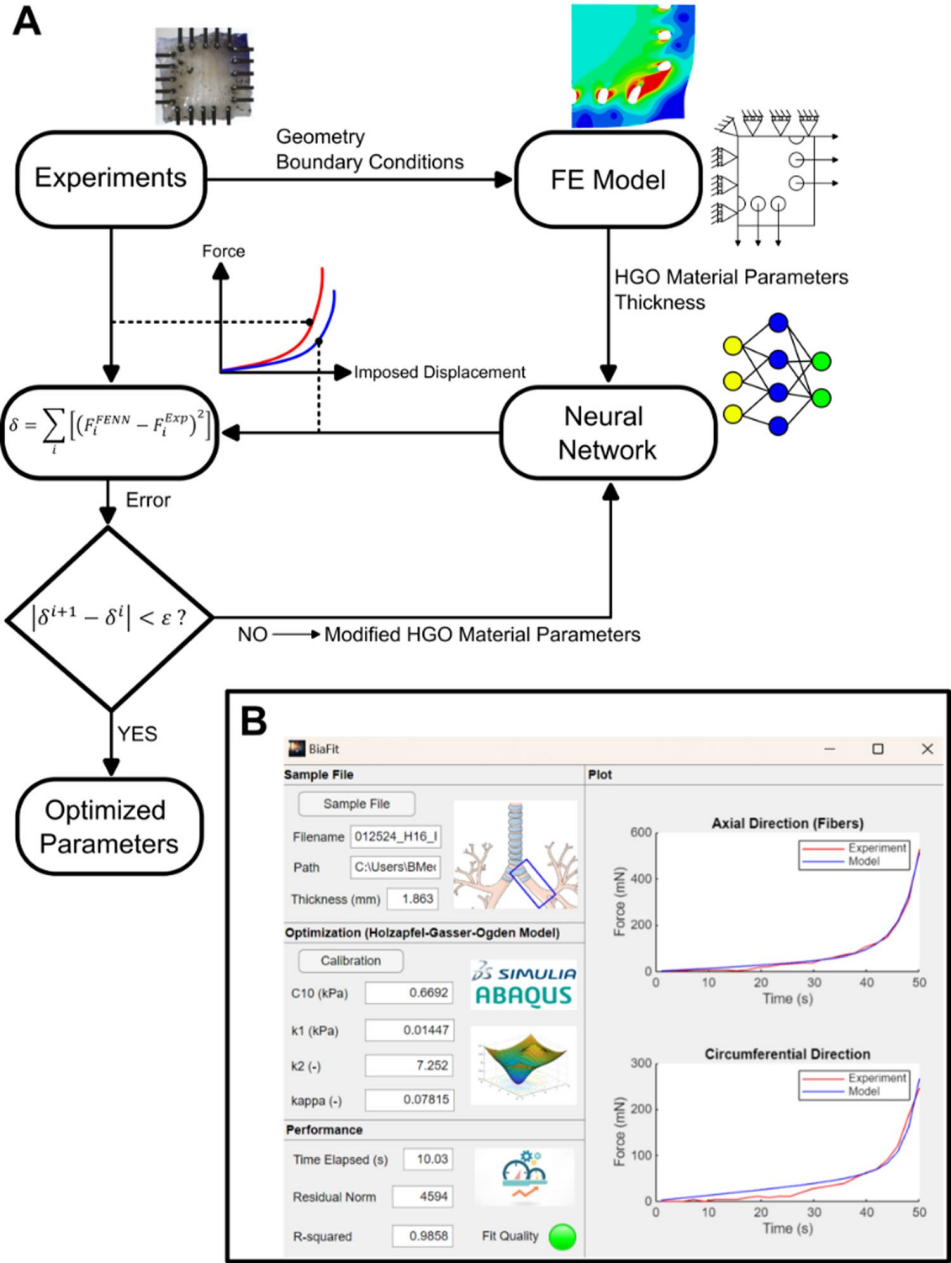
**A** General workflow for characterizing the material parameters of human airway tissue using an inverse finite element and neural network framework. **B** A GUI allows the user to input the experimental file from biaxial testing, and the application then provides the calibrated material properties along with the corresponding fit

An overview of the experimental and numerical pipeline is presented in Fig. 1A. Biaxial tensile experiments were conducted, and then, a FE model was developed to represent the airway samples during testing, incorporating geometry and boundary conditions. To accelerate material parameter identification, a NN surrogate model was constructed and trained using FE simulations with varying material properties. The NN provided instantaneous force predictions for imposed displacements, which were then compared with experimental force data. To optimize the material parameters, an inverse problem was formulated and solved through an optimization procedure, where an objective function was defined to minimize the discrepancy between the model predictions and the experimental results. Finally, a graphical user interface (GUI) was developed (Fig. 1B), allowing users to efficiently characterize human airway samples without running costly FE simulations. The following sections describe each of these steps in-depth, including a brief overview of the biaxial experiments performed on human airway samples, the development of the FE model representing the biaxial test, and the construction and application of the NN in an inverse framework to calibrate material parameters. Finally, a MATLAB application (*BiaFit*) was developed to allow users to apply the trained neural network and characterize HGO material parameters. The application is available at: https://www.mathworks.com/matlabcentral/fileexchange/181573-biafit.

### Experimental biaxial tensile testing of human airways

**Fig. 2:**
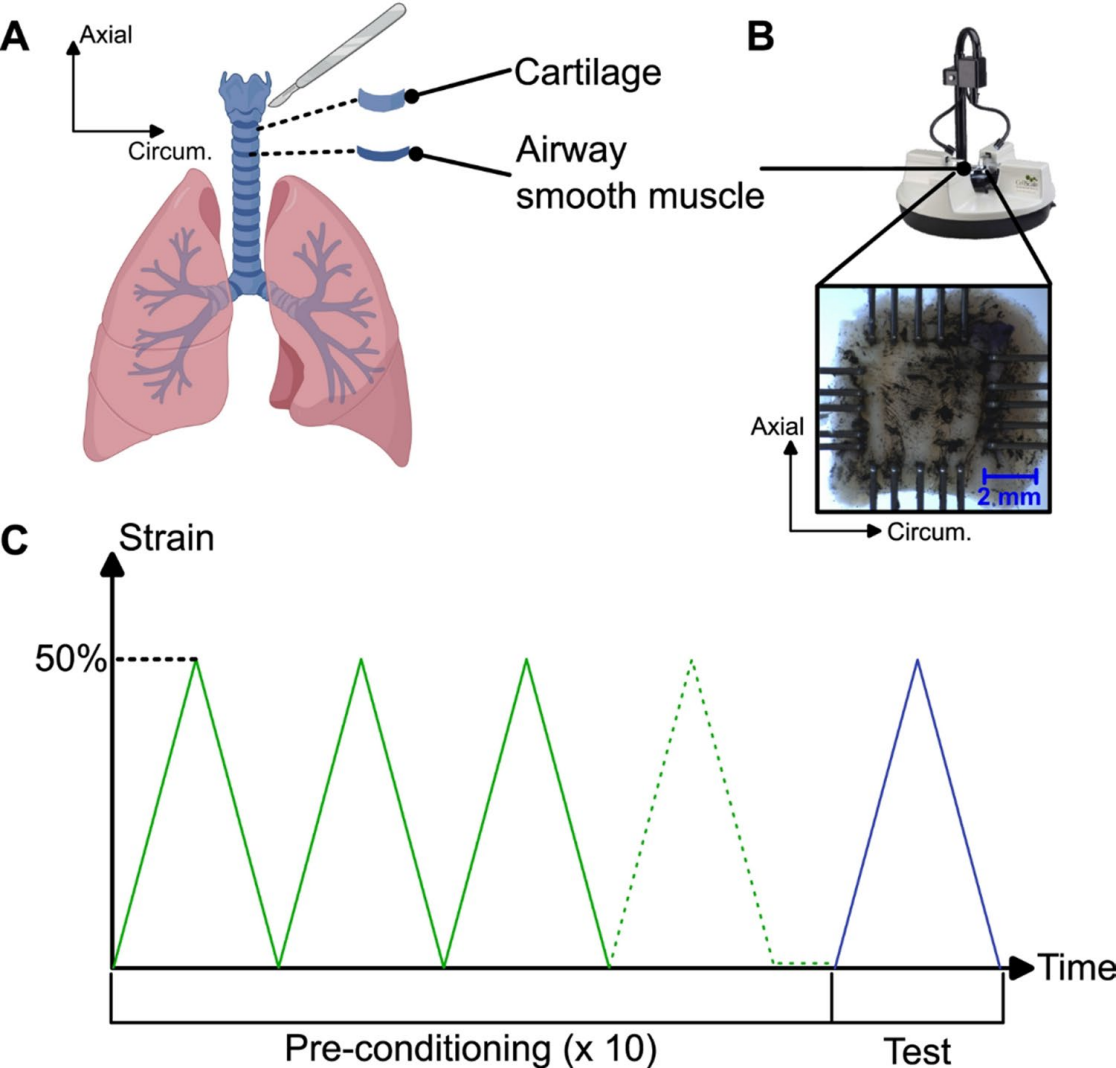
**A** Airway smooth muscle tissue was extracted from each sample (created with BioRender.com). **B** The tissue was mounted on a biaxial tensile testing system and secured using metal rakes. **C **Ten preconditioning cycles (green) were applied to achieve a steady and repeatable the mechanical response before the final test (blue) was recorded

Seven transplant-eligible lung donors were obtained (IRB exemption approval HS 20-180; some specimens were also previously utilized (Quiros et al. 2026; Nelson and Eskandari 2026)), having been deemed healthy enough for clinical use but then reallocated for research purposes due to logistical issues or declining lung function. Airway tissue from each lung was dissected and tested within 48 h of surgical retrieval, as provided by the transplant site. The parenchyma was removed, leaving only the trachea, left primary bronchus, and right primary bronchus. The availability and condition of donor lungs limited the regions that could be consistently sampled across all specimens. As a result, tracheal samples were collected from the region immediately above the carina, which was the only tracheal segment reliably accessible and suitable for mechanical testing from all donors. Where feasible, uniform soft smooth muscle tissue square samples (5.2 × 5.2 mm) were cut away from the cartilage of each airway region (N = 1–3), yielding a total of 46 tissue samples from the seven lungs. Typically, approximately two samples were available per subject per the different airway regions. Each sample was mounted on a biaxial tensile testing system (CellScale Biomaterials Testing, Canada) equipped with 5N load cells and secured using metal rakes (Fig. 2) (Nelson et al. 2024). Samples were extracted from their native tissue immediately before testing. Sample hydration during testing was maintained using 1X phosphate-buffered saline (PBS). Planar equi-biaxial tensile tests were performed on all samples to ensure consistent and controlled loading across various proximal and distal airway regions, while minimizing the risk of tissue damage in smaller bronchial specimens. Samples were stretched to a maximum strain of 50% at a rate of 2.5%/s (Ramirez et al. 2024). Although this strain exceeds typical physiological airway strains (8–31%) (Zhao et al. 2021), it was chosen to allow characterization of both the low-strain response and the transition to higher-strain behavior, while avoiding tissue failure, providing a more thorough mechanical characterization. During protocol development, strains above 60% consistently led to tissue damage near the attachment points. Prior to data collection, each sample underwent 10 preconditioning cycles to establish a consistent reference state. Force–displacement data were then recorded for analysis. Details regarding experimental protocol development and established methodological pipelines, including specific to human airways, can be found in previous studies (Mariano and Eskandari 2024; Nelson et al. 2024; Mariano 2025; Eskandari et al. 2018; Sattari et al. 2023; Mariano et al. 2023).

### FE model construction

**Fig. 3:**
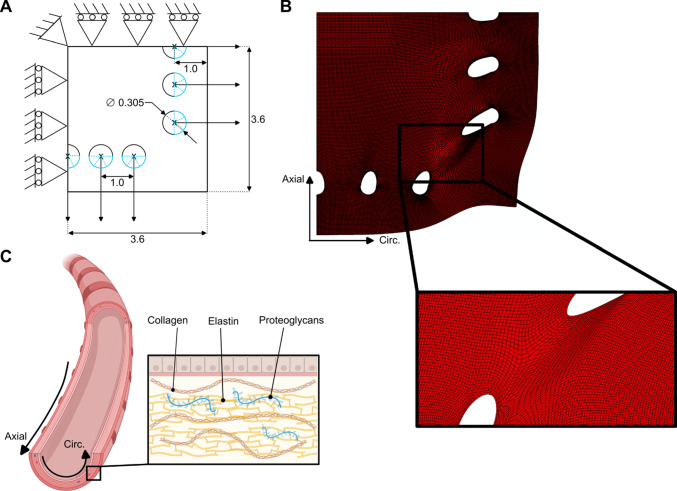
**A** Dimensions of the FE model of the tissue under tensile testing (holes = rake placement) with the applied boundary conditions. **B** Mesh visualization, including a close-up of the region expected to undergo significant deformation during the biaxial test. **C** Schematic representation of an airway, its wall microstructure, and axial and circumferential orientations (created with BioRender.com)

A FE model was developed in ABAQUS (Dassault Systèmes, France) to simulate the biaxial tensile test under quasi-static conditions. The planar dimensions of the model are shown in Fig. 3. The rake diameters and inter-rake distances were set based on manufacturer specifications, while the apron distance (space between rakes and sample edge) was measured across multiple samples, averaging 1.0 ± 0.2 mm. To reduce the computational cost, symmetry was leveraged, and only one-quarter of the FE model was represented. The model thickness was adjusted to match each specific sample. The rakes were modeled indirectly as holes with the same diameter. A reference point was assigned at the center of each hole, and a kinematic constraint was applied between this reference point and the nodes along the semicircular edge (shown in blue in Fig. 3) to simulate rake movement, in accordance with past studies (Fehervary et al. 2016). The experimental displacements prescribed during the biaxial test (Fig. 2) were imposed on the center nodes, while symmetric boundary conditions were applied.

The importance of accurately modeling boundary conditions in biaxial testing has been widely emphasized in the literature (Jacobs et al. 2013). To validate our approach, a preliminary analysis was conducted comparing two models (see Appendix 1): one assuming a simple square sample with uniform displacements applied along its edges and another incorporating the rake representation. The comparison revealed notable differences between the two, highlighting the importance of precise boundary condition representation and supporting the validity of our modeling approach.

The model was meshed with 28,920 quadratic membrane elements, assuming plane stress conditions, a standard approach for modeling thin biological tissues (Kroon 2011; Fehervary et al. 2016, 2019). A mesh convergence analysis was conducted to ensure accuracy, with details provided in Appendix 2.

The Holzapfel–Gasser–Ogden (HGO) material model was used to describe the mechanical behavior of the tissue, as it effectively captures the stress–strain response of anisotropic biological tissues (Gasser et al. 2006; Nolan et al. 2014). The HGO model was defined by the strain energy function $U$ as follows:

1 $$U= {C}_{10}\left({\overline{I} }_{1}-3\right)+\frac{{k}_{1}}{2{k}_{2}}\left[{e}^{{k}_{2}\left(\kappa \left({\overline{I} }_{1}-3\right)+\left(1-3\kappa \right){\left({\overline{I} }_{4}-1\right)}^{2}\right)}-1\right]$$

where ${C}_{10}$, ${k}_{1}$, ${k}_{2}$, and κ are material parameters and κ drives fiber dispersion and is zero when fibers are perfectly aligned in the axial direction (Fig. 2). ${\overline{I} }_{1}$ and ${\overline{I} }_{4}$ represent the first and fourth invariants of the deviatoric Cauchy–Green strain tensor, respectively. The invariants are defined as:

$$\begin{aligned} \overline{I}_{1} & = {\mathrm{tr}}\left( {{\overline{\mathbf{C}}}} \right) \\ \overline{I}_{4} & = {\mathbf{a}}_{0} \cdot {\overline{\mathrm{C}}}{\mathbf{a}}_{0} \\ \end{aligned}$$

where $\overline{\mathbf{C} }={J}^{-2/3}\mathbf{C}$ is the deviatoric right Cauchy–Green tensor, $\overline{\mathbf{C} }={\mathbf{F}}^{T}\mathbf{F}$, $J=\mathrm{d}\mathrm{e}\mathrm{t}(\mathbf{F})$, and ${\mathbf{a}}_{0}$ is the unit vector defining the mean fiber direction in the reference configuration. In the model, the fibers were assumed to be primarily aligned in the axial direction (Fig. 3), following experimental observations (Mariano and Eskandari 2024; Eskandari et al. 2019).

### IFEA calibration

The FE model was first used in an IFEA to estimate material properties for nine samples serving as references, randomly selected from the 46 available samples (three from each of the three airway regions). This analysis helped define the parameter space of interest for training an NN surrogate model. In the second approach, a NN was trained within this parameter space and then used to identify the material properties of all 46 samples.

The IFEA was conducted using *lsqnonlin* in MATLAB (MathWorks, USA), estimating the material parameters of HGO model (Eq. 1): ${C}_{10}$, ${k}_{1}$, ${k}_{2}$, and *κ* (Maghsoudi-Ganjeh et al. 2010b). The analysis was performed separately for samples taken from the trachea, left primary bronchus, and right primary bronchus. The initial parameter ranges (Table 1) were determined through preliminary trial-and-error analyses exploring different orders of magnitude and assessing their qualitative influence on the force–displacement response and were further informed by existing literature on soft biological tissues (Trabelsi et al. 2010; Canales et al. 2023; Nolan et al. 2014). These ranges were selected to ensure numerical stability and parameter identifiability within the inverse finite element framework. Parameter values or combinations outside these ranges frequently led to poor convergence or divergence of the numerical simulations, preventing reliable inverse identification. Accordingly, the bounds should be interpreted as model- and protocol-specific limits.

**Table 1:** Material parameters associated with the HGO model and used in the IFEA. Initial values correspond to the initial guesses used in the lsqnonlin optimization, and lower and upper values define the parameter bounds

Parameters	Initial value	Lower value	Upper value
${C}_{10}$ (kPa)	1.0	$0.01$	$10.0$
${k}_{1}$ (kPa)	0.1	$0.0$	$10.0$
${k}_{2}$ (–)	1.0	$0.0$	$10.0$
$\kappa$ (–)	0.0	$0.0$	$0.33$

The objective of the IFEA was to minimize the difference between experimental and numerical outputs, specifically the force values (Eq. 2). The objective function, denoted as $\varphi$, was expressed as:

2 $$\varphi =\sum_{i=1}^{n}{\left[W({F}_\mathrm{exp})\left({F}_{exp}^{i}-{F}_\mathrm{num}^{i}\right)\right]}^{2}$$

where ${F}_\mathrm{exp}$ and ${F}_\mathrm{num}$​ represent the experimental and numerical force values, respectively. Forces from both loading directions of the biaxial test were included in the objective function. The index $i$ represents each experimental data point used in the fitting procedure. To account for sensor noise and differences in force magnitudes, a weighting factor $W$ was introduced:

3 $$W = \left\{ {\begin{array}{*{20}l} {2, } \hfill & {{\mathrm{if}}\;\;F_{{{\mathrm{exp}}}} \ge 100 \;{\mathrm{mN}}} \hfill \\ {1, } \hfill & {{\mathrm{if}}\;\;F_{{{\mathrm{exp}}}} \ge 30\; {\text{mN and}} \;F_{exp} < 100\;{\mathrm{mN}}} \hfill \\ {0,} \hfill & {{\mathrm{otherwise}}} \hfill \\ \end{array} } \right.$$

This weighting scheme prioritizes more reliable force values while reducing the influence of noisy measurements below 30 mN. Additionally, forces exceeding 100 mN are given greater emphasis, as higher-magnitude values tend to be more accurate (Nelson et al. 2024). A total of n = 50 values were analyzed (as test force values were recorded every 2 s, resulting in 25 values per circumferential and axial direction), and this number of data points ensured an accurate representation of the features of the force–displacement curve. To improve the robustness of the optimization process, the *MultiStart* option with three starting points was used to search for a global minimum. The first starting point corresponds to the initial parameter values reported in Table 1, while the two additional starting points were automatically generated by the *MultiStart* algorithm within the prescribed parameter bounds (The MathWorks Inc. 2022). The convergence criteria were based on those effectively employed in a previous IFEA study (Badrou et al. 2025).

### Neural network and characterization of human airway samples

From the nine sets of calibrated material properties obtained through IFEA using the described FE model, the parameter space for training the NN was refined. This refinement involved narrowing the ranges of the four material properties based on the calibrated values and incorporating sample thickness ($t$) as an additional parameter, with its minimum and maximum values determined from measurements of the 46 available samples (Table 2).

**Table 2:** Parameter space used to train the neural network (NN)

Parameters	Lower value	Upper value
${C}_{10}$ (kPa)	$0.01$	$2.0$
${k}_{1}$ (kPa)	$0.005$	$1.0$
${k}_{2}$ (–)	$1.0$	$10.0$
$\kappa$ (–)	$0.0$	$0.10$
$t$ (mm)	1.24	2.84

A feedforward NN was constructed using MATLAB’s *fitnet* function (The MathWorks Inc. 2022). The input vector of the neural network consisted of the four HGO material parameters (${C}_{10}$, ${k}_{1}$, ${k}_{2}$, $\kappa$) and the sample thickness $t$. The output vector consisted of the force–displacement curves predicted by the neural network, which was trained using FE simulations under prescribed biaxial loading. The NN architecture consisted of two hidden layers with 128 and 64 neurons, respectively, guided by recommendations in the literature (Stathakis 2009; Uzair and Jamil 2020). Training was conducted using 2,000 FE simulations generated via the Latin Hypercube Sampling method (Ye 1998), ensuring comprehensive coverage of the parameter space. The number of snapshots and neurons in each layer were optimized through iterative experimentation, as detailed in Appendix 3.

To enhance the NN’s ability to model complex relationships, *tansig* activation functions were used in the hidden layers, while a linear function was applied to the output layer (Vogl et al. 1988). Bayesian regularization was implemented to mitigate overfitting, ensuring the robustness and generalizability of the NN (MacKay 1992; Burden and Winkler 2008). The training of the neural network was stopped when any of the following conditions were met: (i) the mean squared error (MSE) between the predicted and actual values reached $1{e}^{-7}$, (ii) the gradient of the loss function, which indicates the rate of error reduction when adjusting model parameters, fell below $1{e}^{-4}$, or (iii) the maximum number of 1000 epochs was reached.

The NN was validated through two approaches: (i) retrieving results for the nine reference samples to verify its effectiveness in inverse problems for characterizing HGO material properties and (ii) comparing NN predictions with a batch of 200 new FE simulations generated using Latin hypercube sampling within the prescribed parameter bounds (Table 2), and distinct from the 2,000 FE simulations used for training. The comparison was conducted using the normalized root mean square error (NRMSE in %; Eq. 4) to assess accuracy on unseen data from the 200 new samples:

4 $$\mathrm{NRMSE} \left(\%\right)=\sqrt{\frac{\sum_{j=1}^{m}{({F}_\mathrm{NN}^{j}-{F}_\mathrm{FE}^{j})}^{2}}{\sum_{j=1}^{m}{{(F}_\mathrm{FE}^{j})}^{2}}}\times 100$$

where ${F}_\mathrm{NN}$​ and ${F}_\mathrm{FE}$​ represent the force predicted by the NN and the FE model, respectively. The index $j$ corresponds to each force value along the force–displacement curves, and $m$ is the total number of force values across the 200 samples, i.e., $m=200\times 50=10,000$.

Once validated, the NN replaced the FE model in the IFEA process, enabling the calibration of all 46 samples with significantly reduced computational cost. During post-processing, metrics were employed to assess the quality of the fit for each sample, including the residual norm (sum of squared differences between predicted and experimental forces (The MathWorks Inc. 2022)), and the coefficient of determination (${R}^{2}$), computed as:

5 $${R}^{2}=1-\frac{\sum_{k=1}^{n}{\left({F}_\mathrm{exp}^{k}-{F}_\mathrm{num}^{k}\right)}^{2}}{\sum_{k=1}^{n}{\left({F}_\mathrm{exp}^{k}-\overline{{F }_\mathrm{exp}}\right)}^{2}}$$

where $\overline{{F }_\mathrm{exp}}$ represents the mean of the experimental force values and the index $k$ corresponds to each force value along the force–displacement curves. Additionally, the computational time required for each optimization was recorded, to highlight the efficiency of the NN-based approach.

## Results

### Reference samples: FE simulations and material calibration

**Fig. 4:**
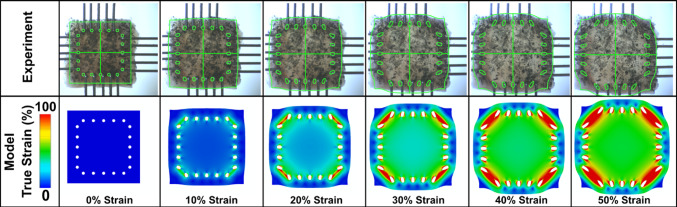
Biaxial tensile test of a representative sample. Top row: experimental images with the corresponding finite element model edges overlaid in green, showing good agreement between experiment and model geometry. Bottom row: numerically predicted true strain magnitude maps for the same loading states, illustrating the spatial distribution of strain within the sample

Using two Intel Xeon 2.10 GHz CPUs, the FE model required an average of one hour to compute for each sample. The primary challenge was handling the high strains induced by displacement and the rakes, which caused localized tissue stretching. The model's performance is illustrated in Fig. 4, where it is qualitatively compared to one of the experimental samples at different time steps.

**Fig. 5:**
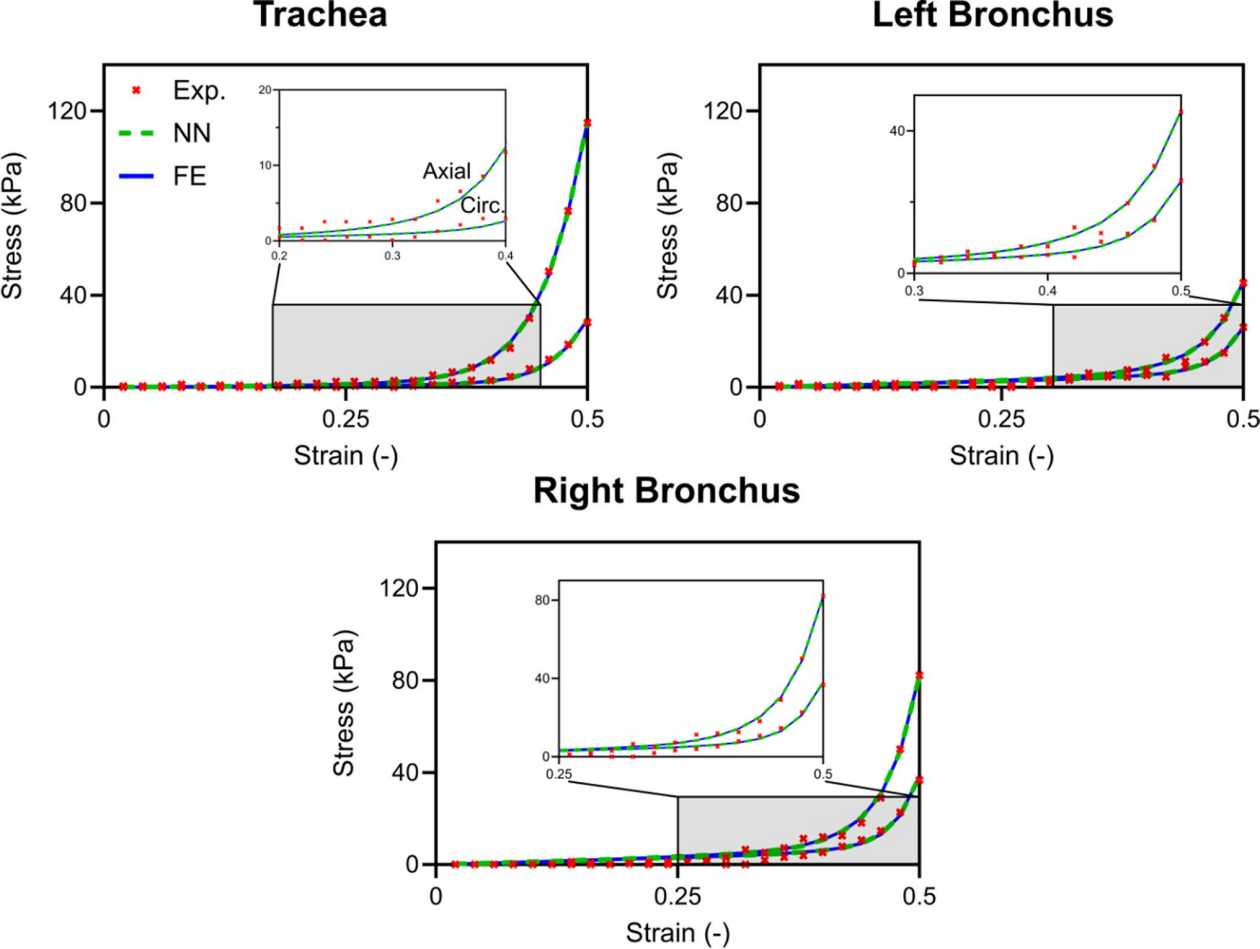
Stress–strain curves for representative samples of the trachea, left bronchus, and right bronchus selected from the nine reference samples (trachea #3, left bronchus #1, and right bronchus #1; see Table 3). Results are shown for both axial and circumferential directions after material calibration using the neural network (NN) surrogate and the finite element (FE) model

Using the FE model, IFEA was performed for the nine reference samples. All three optimization runs converged to an identical solution, supporting the robustness of the inverse procedure. The computational cost for each sample was approximately one day. The optimized HGO material parameters are reported in Table 3, and representative stress–strain curves fitted to the experimental data are shown in Fig. 5. Engineering (first Piola–Kirchhoff) stress was computed from the measured force using the specimen thickness and the effective testing area, while strain was defined as the elongation divided by the initial length. The remaining fitted curves are provided in Appendix 4. It can be observed that the FE model, combined with the integrated HGO material law, successfully captured the complexity of the curve, which is characterized by a bilinear response, where the second portion typically exhibited a high peak value. The parameter κ, which governs fiber dispersion, converged to a value close to 0, indicating that the fibers are primarily aligned in the axial direction. Based on these reference results, the parameter ranges were refined to train the neural network in a subsequent phase (see Table 2).

**Table 3:** HGO material resultant parameters for the nine reference samples, obtained from IFEA (blue) and NN predictions (green). The coefficient of determination (R^2^), defined in Eq. 5, indicates the quality of fit of both models to the experimental force–displacement data

Region	Sample	${C}_{10}$ (kPa)	${k}_{1}$ (kPa)	${k}_{2}$(–)	$\kappa$(–)	${R}^{2}$
Trachea	#1	0.60 0.59	0.031 0.040	4.9 4.7	0.07 0.07	0.96 0.96
	#2	0.42 0.48	0.841 0.730	3.4 3.6	0.09 0.09	0.99 0.98
	#3	0.12 0.12	0.122 0.121	6.6 6.6	0.07 0.07	0.99 0.99
Left Bronchus	#1	0.54 0.55	0.031 0.030	6.1 6.2	0.08 0.08	0.98 0.98
	#2	0.47 0.48	0.075 0.068	4.5 4.6	0.02 0.02	0.97 0.97
	#3	0.57 0.60	0.036 0.006	3.6 5.1	0.02 0.00	0.95 0.95
Right Bronchus	#1	0.62 0.62	0.014 0.013	8.0 8.2	0.08 0.08	0.98 0.98
	#2	0.90 0.91	0.131 0.100	2.4 2.7	0.09 0.09	0.97 0.09
	#3	0.57 0.67	0.018 0.010	8.1 8.9	0.07 0.07	0.99 0.99

### NN validation

**Fig. 6:**
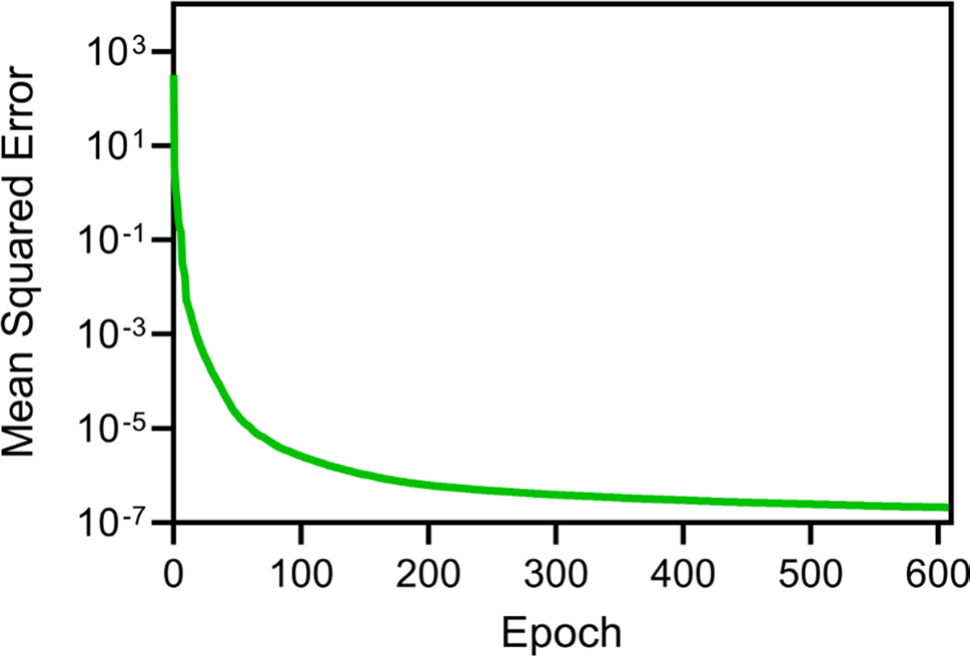
Mean squared error computed between the predicted force values from the neural network and the experimental values during the training phase, plotted against the number of epochs

Using five Intel Xeon 2.10 GHz CPUs, the 2000 FE simulations were completed in approximately 8 days. The NN was then trained for 609 epochs (see Fig. 6; performance vs. number of epochs), achieving an error of 0.9% when compared to the FE model results for the 200 new test samples.

The NN was then used to retrieve the calibrated material parameters via IFEA by replacing the FE model with the NN for the optimization process. The results (blue: FE model; green: NN) are summarized in Table 3. Similar results were observed, except for discrepancies in sample #3 of the left bronchus: the force values for this sample were small and noisy, leading to multiple possible solutions. It was hypothesized that the difference was due to the variation in parameter ranges between the NN and the FE model. To verify this, an IFEA was performed using the same parameter range as the inverse method with the NN and obtained identical results, mitigating concerns.

### NN validation

The NN was subsequently used to characterize the remaining 37 samples. The comprehensive results, including the metrics defined in section “Neural network and characterization of human airway samples”, are provided in Appendix 5.

**Fig. 7:**
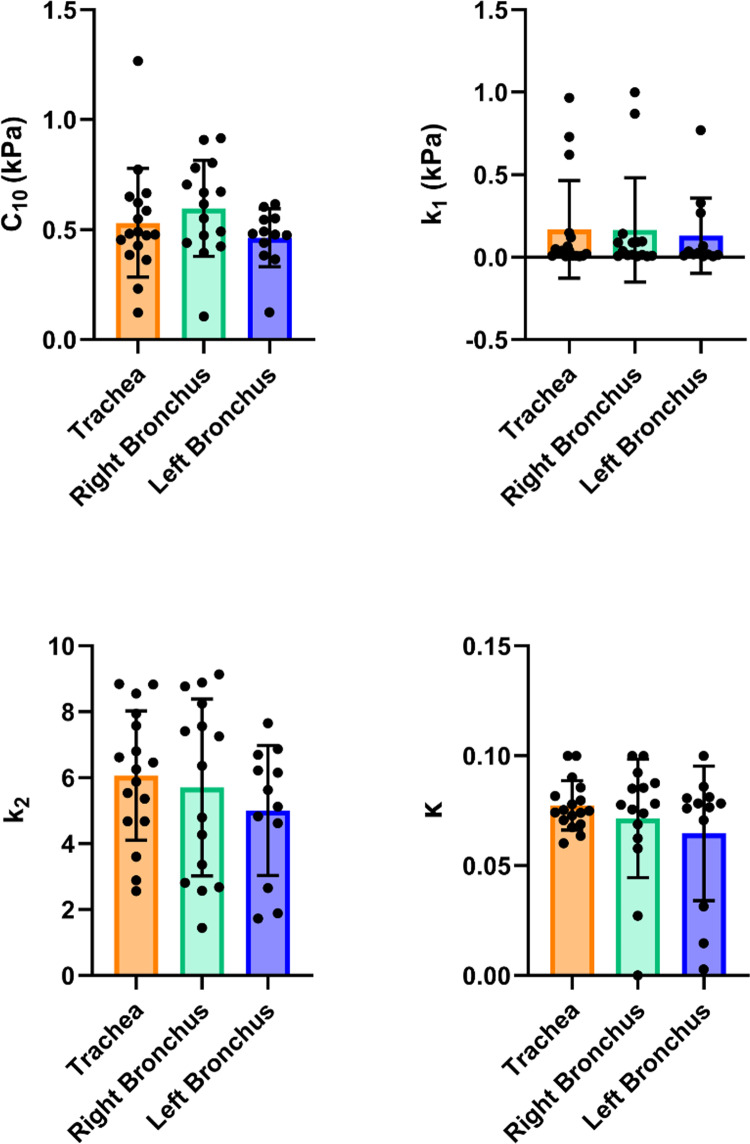
Bar plots showing the calibrated HGO material parameters C_10_, *k*_1_, *k*_2_, and κ for the trachea, right primary bronchus, and left primary bronchus. Individual data points are shown together with group means ± standard deviation. Regional differences were assessed and no statistically significant differences were observed between airway region parameters

Table 4 summarizes the calibrated HGO material parameters for the trachea, left primary bronchus, and right primary bronchus, reported as mean ± standard deviation. Figure 7 presents the comparison of these parameters across airway regions. Regional differences were assessed using one-way ANOVA with Bonferroni post hoc correction. No statistically significant differences were observed between regions for any of the fitted parameters (all comparisons: *p* ≥ 0.05). Although variations in group mean values were observed, substantial overlap between regions was present for all parameters. And while no significant regional differences were observed, modest variations in group mean values were apparent. In particular, the trachea and right bronchus tended to exhibit higher mean values of $C_{10}$, $k_{1}$, and $k_{2}$ compared to the left bronchus, while the trachea showed a slightly lower mean $C_{10}$ value relative to the right bronchus. These trends should be interpreted cautiously due to substantial overlap between regions.

**Table 4:** Calibrated HGO material parameters for the 46 samples, obtained through optimization. The mean $\pm$ standard deviation values are reported across respective tracheal and bronchial regions

Region	${C}_{10}$(kPa)	${k}_{1}$(kPa)	${k}_{2}$ (–)	$\kappa$ (–)
Trachea	0.53 ± 0.25	0.17 ± 0.30	6.1 ± 2.0	0.08 ± 0.01
Left Bronchus	0.60 ± 0.22	0.17 ± 0.32	5.7 ± 2.7	0.07 ± 0.03
Right Bronchus	0.46 ± 0.13	0.13 ± 0.23	5.0 ± 2.0	0.07 ± 0.03

These numerical results correlated well with experimental trends and bilinear behavior (Fig. 5). The high values of ${k}_{2}$ (5.0 to 6.1) across all regions were influenced by the bilinearity of the material response, where a higher ${k}_{2}$ value suggests a delayed activation of fiber contribution, which aligns with the observed mechanical behavior. The value of the parameter $\kappa$, which governs fiber dispersion, remained relatively low (0.08) indicating strong fiber alignment along the axial direction. This finding supports the hypothesis and past experimental works that collagen fibers are predominantly oriented in the axial direction, playing a key role in the mechanical behavior of airway structures (Mariano et al. 2023).

The total computational cost for calibrating all 46 samples was approximately 6 min, compared to several weeks using the traditional IFEA process (estimated based on results from the nine reference samples). This highlights the necessity of using the surrogate NN in the inverse problem to significantly reduce computational time.

## Discussion

This study presents a comprehensive pipeline to identify the material parameters of the human trachea, right bronchus, and left bronchus smooth muscle using the HGO material model. While direct comparisons with previously reported HGO parameters are limited by differences in constitutive form and experimental loading protocol, Trabelsi et al. reported a matrix shear parameter ${C}_{10}=0.88$ kPa for a for a modified two-fiber-family HGO model fitted to uniaxial human trachea tests (Trabelsi et al. 2010). This magnitude is comparable to our fitted matrix response (${C}_{10}=0.53$ kPa), suggesting a similar order of stiffness for the isotropic smooth muscle matrix. However, Trabelsi et al. reported substantially larger exponential nonlinearity parameters for the fiber families (analogous to ${k}_{2}$; e.g., ${k}_{2}=34$ and ${k}_{4}=14$) than those obtained here (${k}_{2}<10$). This discrepancy is expected given (i) the different model structure (two fiber families versus our parameterization) and (ii) different loading conditions and strain ranges (uniaxial tests versus our biaxial experiments), both of which strongly affect identifiability of the exponential stiffening parameters. We therefore restrict cross-study interpretation primarily to the matrix stiffness scale (${C}_{10}$) and treat the fiber exponential parameters as protocol and model dependent.

More broadly, quantitative characterization of airway soft tissue mechanics remains limited in the pulmonary biomechanics literature, with most existing studies focusing either on the whole composite airway behavior or on bulk parenchymal lung tissue. Only a few studies have directly characterized the mechanical properties of the trachea (Safshekan et al. 2016; Wang et al. 2011; Huang et al. 2021). For example, Huang et al. characterized the trachea as a composite whole in porcine, caprine, and canine models and reported stiffness values ranging from 1.2 to 2.6 MPa, while emphasizing that the dominant contribution to overall stiffness arises from the rigid cartilage rings (Huang et al. 2021; Safshekan et al. 2016). We can derive an effective stiffness from our measured material parameters and compare it with reported values for pulmonary soft tissues. Specifically, from the matrix shear parameter ${C}_{10}$, an effective Young’s modulus $E$ can be estimated assuming incompressibility, such that $E=6{C}_{10}$, yielding $E\approx 3.2$ kPa for the trachea. Although this comparison warrants further investigation, the methods used to quantify stiffness differ from those in the current study. Additionally, Polio et al. reported Young’s moduli for bulk lung tissue ranging from approximately 1 to 6 kPa using multiple characterization techniques, including uniaxial testing (Polio et al. 2018). Although these measurements primarily reflect the mechanical behavior of lung parenchyma rather than airway tissue specifically, they support the plausibility of the stiffness scale obtained in this study.

While alveolar walls and airway tissues differ anatomically, both are composed of compliant, extracellular-matrix-dominated constituents that govern the low-strain mechanical response prior to fiber recruitment. Consistent with this, multiscale modeling studies of lung parenchyma have estimated alveolar wall elastic moduli on the order of 4–6 kPa, with substantially stiffer fibrous components contributing at higher strains (Bou Jawde et al. 2020). Together, these comparisons support the interpretation that the effective small-strain modulus inferred here ($E\approx 3.2$ kPa) represents a physiologically reasonable matrix-level stiffness within pulmonary soft tissues.

Considering the experimental biaxial characterization data only, we further compared our results with a previous study on cartilage-free porcine airways (Sattari et al. 2023). As in prior work, the initial and ultimate moduli were obtained directly from experimental stress–strain curves using linear fits with ${R}^{2}>0.9$ (Smith et al. 2008; Sattari et al. 2023). At 5%/s, porcine tracheal tissues exhibited axial and circumferential initial moduli of $14.1\pm 3.6$ kPa and $38.9\pm 16.2$ kPa, respectively, while the large bronchi were less stiff ($9.1\pm 3.1$ kPa axially and $26.6\pm 9.2$ kPa circumferentially). Using the same procedure, human airway tissues showed comparable magnitudes, with axial initial moduli of $22.3\pm 7.4$ kPa in the trachea and $\thicksim20.1$ kPa in the bronchi (averaged left/right), and lower circumferential initial moduli of $13.2\pm 6.5$ kPa in the trachea and $\thicksim10.0$kPa in the bronchi. Ultimate moduli were substantially higher in both species. At 5%/s, porcine tracheal tissues reached $588.4\pm 335.9$ kPa axially and $367.6\pm 236.5$ kPa circumferentially, compared with $293.4\pm 206.8$ kPa axially and $107.0\pm 31.2$ kPa circumferentially in the bronchi. Human airway tissues exhibited similar magnitudes, with axial ultimate moduli of $592.3\pm 542.1$ kPa in the trachea and $\thicksim403.9$kPa in the bronchi (averaged left/right), and circumferential ultimate moduli of $284.1\pm 355.3$ kPa in the trachea and $\thicksim145.4$ kPa in the bronchi.

To more efficiently calibrate material parameters compared to computationally-intensive traditional IFEA methods, the NN was implemented as a surrogate model within the optimization framework. This approach, relying on built-in MATLAB functions, circumvents the need for intrusive methods that embed FE equations directly into the NN (Meethal et al. 2023; Hu et al. 2024). The pipeline was designed with a rigorous methodology, ensuring a smooth transition from biaxial experimental data to parameter identification. By integrating this machine learning approach, the computational burden associated with traditional IFEA was significantly reduced, making large-scale parameter identification feasible (Torun et al. 2022; Badrou et al. 2025).

Ultimately, the improved inverse modeling approach described in this study enhances the accuracy and speed of numerical simulations and will be a useful framework for future computational modeling endeavors. Moreover, this study’s material characterization of tracheal and bronchial tissues offers valuable insights into airway mechanics. These findings have direct implications for optimizing interventions, including stent design and placement, to ultimately reduce complications, such as stent migration, in the case of central airway obstruction due to lung cancer.

### Limitations and future directions

While the results are promising and the proposed framework provides a solid foundation for future developments, several aspects of the study should be considered for refinement in future works. For instance, noisy force measurements at low force levels required the use of a weighting factor in the objective function to improve the robustness of the parameter identification. This weighting scheme was introduced to address experimental and numerical limitations rather than to directly represent physiological loading levels. The force thresholds of 30 mN and 100 mN were selected based on experimental observations, where measurements below 30 mN were strongly affected by sensor noise, while higher force values were more stable and reproducible (Cleveland 1979). Importantly, the applied deformation levels were chosen based on physiological considerations, whereas the weighting factor was used only during the fitting process to prioritize reliable portions of the force–displacement response. Although further refinement of this weighting approach could be explored in future studies, this method was necessary to ensure stable and accurate identification of the material parameters.

Another limitation of this study is the dispersion observed in the experimental data. This variability is mainly related to the difficulty of handling and testing very soft airway tissues, especially when working with small samples (Nelson et al. 2024). Due to their low stiffness and limited dimensions, these samples are highly sensitive to minor variations in handling, gripping, alignment, and boundary conditions, which can introduce uneven pre-strain or small differences in loading and increase variability in the measured response no matter how meticulous the experimental preparation was. In addition, real biological differences between subjects are expected and likely contribute to the observed spread in the calibrated material parameters. From a modeling point of view, this dispersion means that airway tissue properties should not be represented by a single fixed set of parameters. Instead, the reported variability provides useful information on realistic parameter ranges that can be used in computational models. In future work, we aim to reduce this dispersion by increasing the number of samples and by grouping specimens based on subject-specific characteristics.

From a modeling perspective, another limitation of this work is the potential non-uniqueness of the identified HGO material parameters. Like other exponential constitutive models, the HGO formulation can lead to different combinations of parameters producing similar force–displacement responses. In this study, the objective was to identify a consistent and stable combination of parameters that reproduces the experimental response within the considered loading range. To limit non-uniqueness, parameter bounds were imposed based on numerical stability and preliminary analyses, the full force–displacement curve was used in the objective function, and a *MultiStart* optimization strategy was applied.

Validation of the FE model remains a critical step to ensure that the NN is trained on accurate predictive simulations. Nevertheless, it is difficult to define appropriate comparison metrics between numerical and experimental results. Ideally, strain measurements obtained using digital image correlation (DIC) could be used to define realistic deformation levels, as demonstrated in previous studies from our group (Mariano et al. 2020; Quiros et al. 2022; Nelson et al. 2023; Shankel et al. 2025a, b; Quiros et al. 2024; Mariano et al. 2022). However, in the present study, accurate DIC strain measurements during biaxial testing were difficult to obtain. This was due to the rapid tissue deformation, combined with the small sample dimensions and limitations in speckle pattern quality, which ultimately reduced the reliability of the DIC-based strain measurements. As a result, despite collecting the data, strains from DIC were not used beyond qualitative assessment in the neural network framework. As a result, strain data were not used in the neural network framework, and force–displacement responses were instead selected as the primary outputs. Future work will focus on improved optical setups and imaging strategies to enable reliable strain-based inputs.

Geometric variability across samples posed another limitation in the modeling process. Rake dimensions and planar geometry were based on standard documentation, while thickness was measured experimentally and incorporated into the model. However, differences in inter-rake spacing and apron dimensions between samples were not accounted for, potentially affecting the resulting material parameters (Pei et al. 2021). Future work should explore sample-specific modeling techniques using imaging data to generate subject-specific geometries (Fehervary et al. 2019) and assess the influence of the apron (Di Leonardo et al. 2024).

Additionally, a plane-stress assumption was adopted when modeling the airway samples. However, the ratio of sample length to thickness is below the commonly accepted threshold of 10:1 and represents a limitation of the present study. Nevertheless, the use of plane stress was motivated by prior studies employing similar approaches (Fehervary et al. 2016, 2019) and its common use in the modeling of biological tissue samples (Holzapfel and Weizsäcker 1998; Kroon 2011). Future work should investigate the impact of this assumption by developing full 3D models of the biaxial test. In parallel, experimental strategies aimed at reducing sample thickness could help better satisfy plane-stress conditions.

The choice of the HGO material model was motivated by the nonlinearity of lung tissue. While the model provided a good fit, it may not fully capture the whole complexity of airway mechanics (Eskandari et al. 2019; Maghsoudi-Ganjeh et al. 2021a). Lung tissue exhibits not only anisotropic but also heterogeneous and viscoelastic behavior (Eskandari et al. 2018; Bayliss and Robertson 1939; Hurtado et al. 2017; Amelon et al. 2011; Nelson et al.^2025^; Sattari et al. 2020). Incorporating viscoelastic experimental data into the pipeline would require modifications to the material formulation, likely increasing the number of parameters and, consequently, the computational cost of the FE model—however the tradeoff may well be warranted.

Further improvements must also be made on the machine learning approach. The current study relied on a feedforward neural network, but advancements in deep learning, such as recurrent neural networks, could enhance predictive capabilities (Shi et al. 2024; Tao et al. 2024). Emerging techniques that bypass the computational expense of FE simulations for training surrogate models could also be integrated into this pipeline (Motiwale et al. 2024).

Finally, the current pipeline was developed using lungs from transplant-grade specimens that were rejected due to logistical reasons or reduced lung function and were therefore designated for research use. Importantly, these lungs were not obtained from patients with severe lung disease and were considered representative of relatively healthy tissue. As a result, this study does not capture the mechanical behavior of diseased airways. Extending the present pipeline to include pathological cases is an important next step, as airway material properties are known to change significantly in disease (Strohl et al. 2012; Williamson et al. 2011). Such extensions would also strengthen the connection with clinical applications, particularly for patients with respiratory diseases who require airway stenting procedures.

In addition, the material properties reported in this study were obtained from cadaveric lung specimens tested within 48 h post mortem and therefore may differ from in vivo physiological conditions. Post-mortem changes, the absence of smooth muscle tone, lack of perfusion, and differences in loading environment may influence the measured mechanical response.

## Conclusion

In this study, we developed a numerical pipeline to identify the mechanical properties of human airway tissue. Biaxial tensile testing is combined with an inverse finite element analysis (IFEA) procedure, where the optimization process is then improved with construction of a neural network (NN) surrogate model to allow highly efficient parameter identification. By replacing the computationally expensive traditional IFEA approach with the trained NN in the inverse optimization process, we significantly reduced the time required for material characterization, while maintaining accuracy. The obtained material parameters provide a refined understanding of airway biomechanics, addressing an important knowledge gap in the literature. The findings not only improve current airway models but also represent a step forward in the development of predictive computational frameworks for respiratory medicine. These models could be integrated into clinical applications, such as patient-specific simulations for airway interventions, stent design optimization, and surgical planning, ultimately contributing to improved treatment strategies for airway-related conditions. Furthermore, the methodology and graphical interface can readily be extended to study airway tissues across different species or adapted for other soft biological tissues, such as brain, skin or arterial structures, broadening its applications in computational biomechanics.

## Supplementary Information

Below is the link to the electronic supplementary material.

Supplementary file1 (DOCX 2153 kb)

Supplementary file2 (DOCX 18 kb)

Supplementary file3 (DOCX 19 kb)

Supplementary file4 (DOCX 907 kb)

Supplementary file5 (XLSX 21 kb)

## Data Availability

Considering the restrictions and nature of the human tissues involved in this study, the raw data supporting the conclusions of this article will be made available by the authors, upon reasonable request.
