# Supplementary material for: Human airway material characterization via inverse finite element analysis and neural network surrogate
Source: Biomech Model Mechanobiol. 2026 Jun 3;25(3):61. doi: 10.1007/s10237-026-02081-7 (PMC13233878; doi:10.1007/s10237-026-02081-7)
Supplement: Supplementary file 1 — Supplementary file1 (DOCX 2153 kb) [file 10237_2026_2081_MOESM1_ESM.docx]

**Impact of Defining Correct Boundary Conditions: Example from Biaxial Experiment Simulations Using the Holzapfel-Gasser-Ogden (HGO) Model**

The objective of this study was to evaluate the impact of accurately representing boundary conditions when simulating biaxial experiments using the HGO model. To perform this analysis, we constructed two geometries (Fig. 1): one representing the sample with holes for the rakes and another considering only the central square, where displacements were applied at the edges instead of the hole centers, as discussed in the main content of the article.


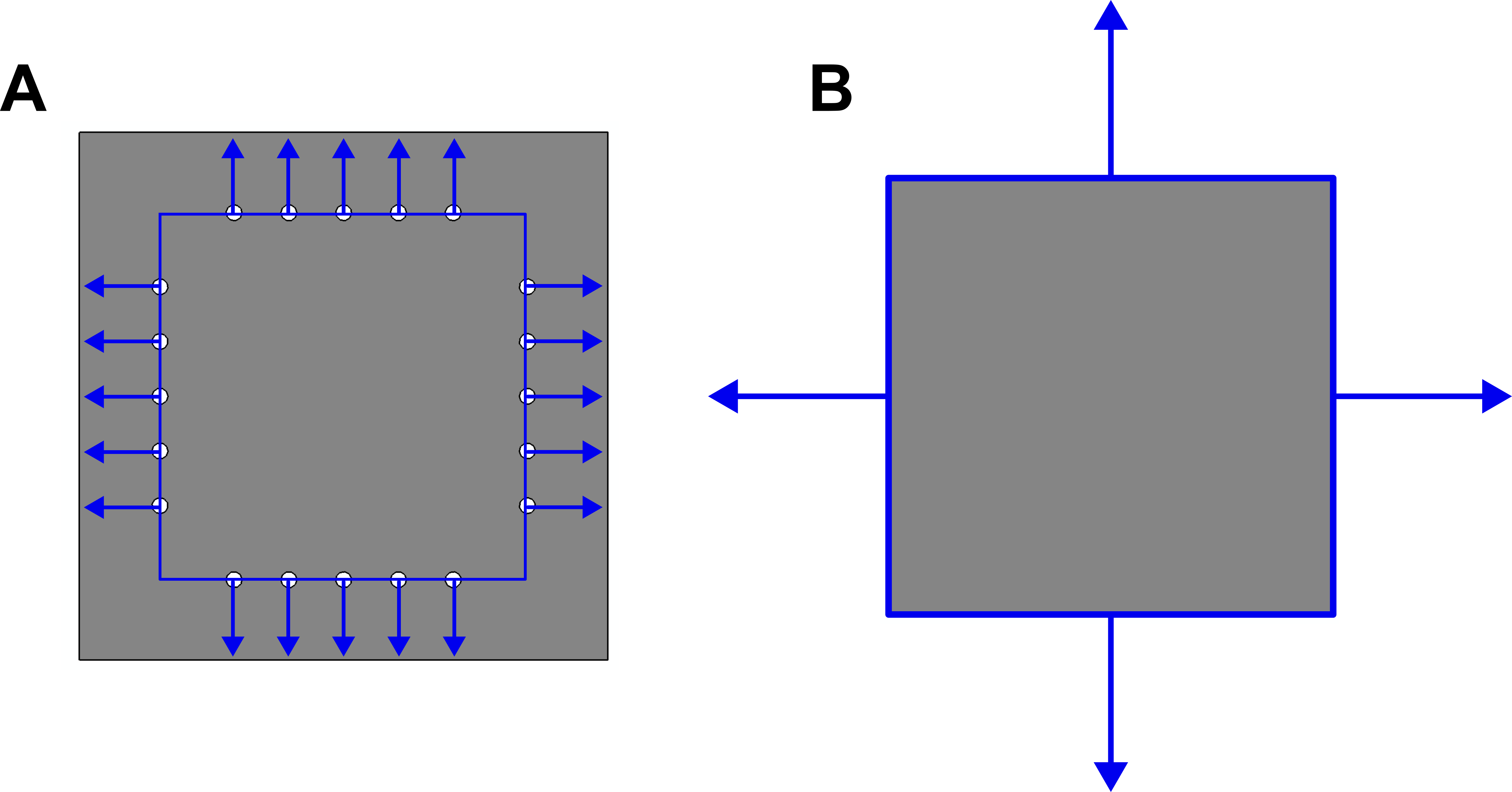


Figure 1: **(A)** Biaxial model representing the rake holes and **(B)** simplified model geometry, considering only the central region of interest inside the rakes. Blue arrows indicate the direction of displacement.

We tested four different material sets (Table 1) and compared the force-displacement curves in both directions (Fig. 2). Notable differences were observed between the model with rakes and the square model.

Table 1: Set of material parameters for the different test cases, comparing analyses with and without rakes.

| **Test** | $\boldsymbol{C}_{\boldsymbol{10}}$ **(kPa)** | $\boldsymbol{k}_{\boldsymbol{1}}$ **(kPa)** | $\boldsymbol{k}_{\boldsymbol{2}}\boldsymbol{(-)}$ | $\boldsymbol{\kappa(-)}$ |
| --- | --- | --- | --- | --- |
| **#1** | 1.0 | 1.0 | 1.0 | 0.1 |
| **#2** | 10.0 | 10.0 | 1.0 | 0.1 |
| **#3** | 100.0 | 1.0 | 1.0 | 0.1 |
| **#4** | 10.0 | 10.0 | 1.0 | 0.33 |


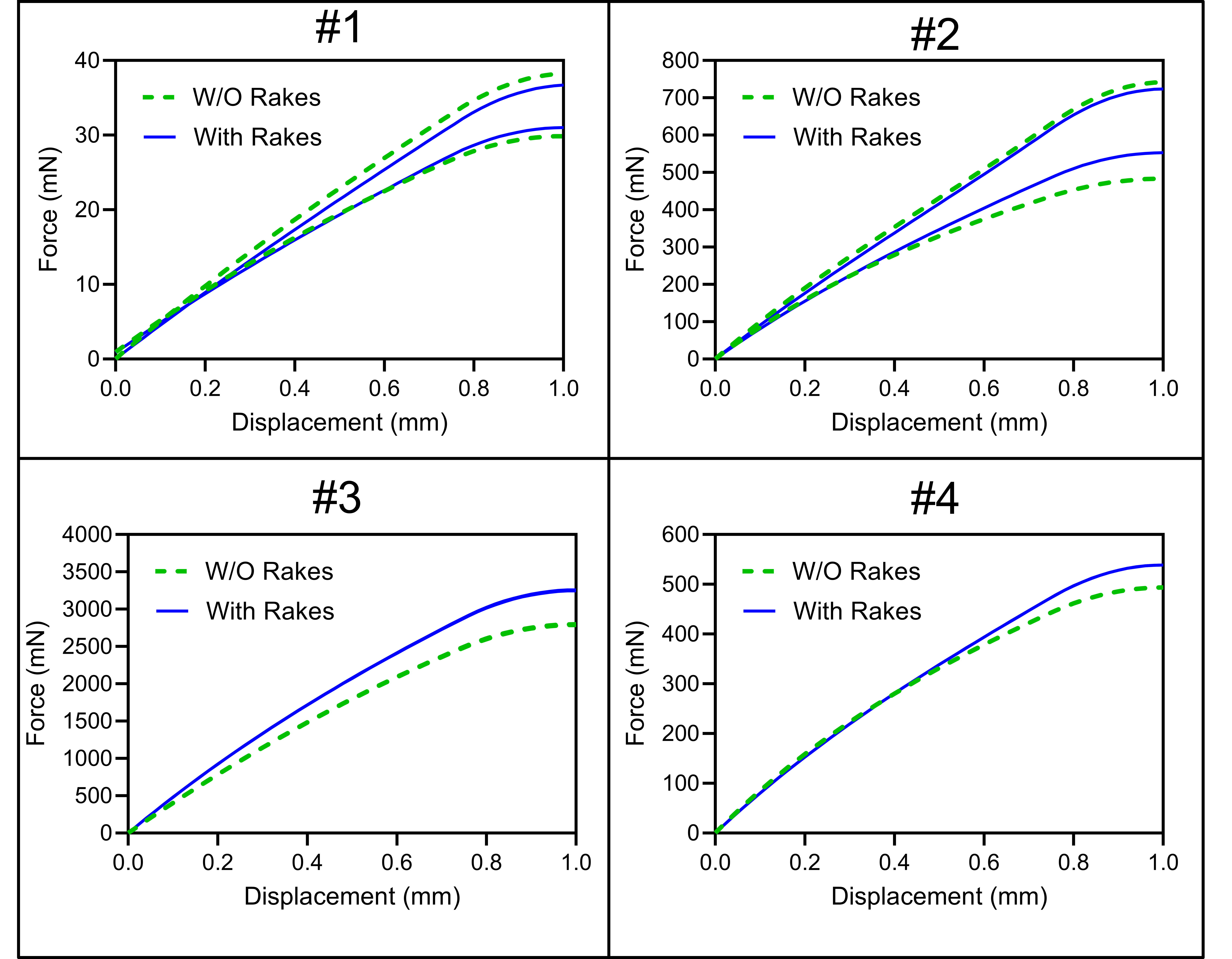


Figure 2: Results comparing the models with and without rakes for the four test cases (referencing Table 1) show that higher force values correspond to the axial (fiber) direction. The differences between the two directions for samples #3 and #4 are not prominent, as the values are closely matched.
