# Supplementary material for: Human airway material characterization via inverse finite element analysis and neural network surrogate
Source: Biomech Model Mechanobiol. 2026 Jun 3;25(3):61. doi: 10.1007/s10237-026-02081-7 (PMC13233878; doi:10.1007/s10237-026-02081-7)
Supplement: Supplementary file 2 — Supplementary file2 (DOCX 18 kb) [file 10237_2026_2081_MOESM2_ESM.docx]

**Mesh Convergence Analysis for Biaxial Simulation**

The objective of this study was to perform a mesh convergence analysis to determine the optimal number of elements for the finite element (FE) model simulating biaxial experiments. The model used for this analysis was similar to the one described in the main document, with a thickness of 2 mm and the following material parameters for the HGO model: $C_{10}=10$ kPa, $k_{1}=10$ kPa, $k_{2}=1.0$, and $\kappa=0.1$. Displacement was applied in the same manner at 50% strain, and the maximum force value was recorded for each test case. To select the appropriate number of elements, we considered CPU time, peak force values compared to test cases with a fine mesh, and mesh quality constraints that could limit deformation during the simulation. The results are presented in Table 1.

Table 1: Results of the mesh convergence analysis. The bolded line indicates the selected configuration for the final FE model.

| **Test Case** | **Number of Elements** | **CPU Time (s)** | **Maximum Force (mN)** |
| --- | --- | --- | --- |
| **1** | 1,269 | 58 | 1430 |
| **2** | 3,475 | 141 | 1420 |
| **3** | 12,752 | 439 | 1417 |
| **4** | **28,920** | **911** | **1417** |
| **5** | 116,053 | 3,750 | 1417 |
| **6** | 469,867 | 18,839 | 1418 |
