# Supplementary material for: Human airway material characterization via inverse finite element analysis and neural network surrogate
Source: Biomech Model Mechanobiol. 2026 Jun 3;25(3):61. doi: 10.1007/s10237-026-02081-7 (PMC13233878; doi:10.1007/s10237-026-02081-7)
Supplement: Supplementary file 3 — Supplementary file3 (DOCX 19 kb) [file 10237_2026_2081_MOESM3_ESM.docx]

**Analysis to Determine the Optimal Number of Neurons and Training Data for the Neural Network (NN)**

The objective of this study was to describe the methods used to determine the optimal number of neurons for the two layers of the NN and the number of finite element (FE) simulations required for training. The computational resources used for this process were the same as those defined in the main paper: five Intel Xeon 2.10 GHz CPUs. The process followed these steps:

1. We started with 500 FE simulations for training, a number deemed reasonable as it required approximately one day to complete.
2. We trained an NN using the 500 FE samples with two layers and tested different neuron configurations per layer: [32; 16], [64; 32], and [128; 64]. Beyond these neuron counts, the computational cost of training became excessive.
3. We repeated this process for two additional NNs trained with 1,000 and 2,000 FE simulations, resulting in a total of nine NNs.
4. We created three validation batches, each containing 10% of the total FE simulations, leading to validation sets of 50, 100, and 200 FE simulations, respectively.

An error was measured between each NN and the three different validation batch using the metric as follows:

$$NRMSE \left( \% \right)=\sqrt{\frac{\sum_{i=1}^{m} {(F_{NN}^{i}-F_{FE}^{i})}^{2}}{\sum_{i=1}^{m} {{(F}_{FE}^{i})}^{2}}}\times100 (1)$$

where $F_{NN}$​ and $F_{FE}$​ represent the force predicted by the NN and the FE model, respectively, and $m$ is the total number of force values across the samples. We finalized the training with an NN using 2,000 samples and 128 and 64 neurons in the first and second layers, respectively, as the validation errors remained below 1%. This error threshold was deemed satisfactory since, at 1%, the force-displacement curves were indistinguishable.

Table 1: Results of the nine NNs compared with the validation sets using Eq. 1. Errors are expressed as percentages.

| **NN 500** | **[128; 64]** | **[64; 32]** | **[32; 16]** |
| --- | --- | --- | --- |
| **Error 200** | 23.4 | 21.2 | 23.2 |
| **Error 100** | 38.2 | 3.4 | 15.9 |
| **Error 50** | 23.1 | 31.1 | 12.8 |
| **NN 1,000** | **[128; 64]** | **[64; 32]** | **[32; 16]** |
| **Error 200** | 28.6 | 17 | 27.9 |
| **Error 100** | 10.4 | 3.5 | 3.8 |
| **Error 50** | 22.6 | 9.6 | 18.3 |
| **NN 2,000** | **[128; 64]** | **[64; 32]** | **[32; 16]** |
| **Error 200** | **0.9** | 5.4 | 5.7 |
| **Error 100** | **0.3** | 2.7 | 3.3 |
| **Error 50** | **0.8** | 5.3 | 3.7 |
