# Supplementary material for: Human airway material characterization via inverse finite element analysis and neural network surrogate
Source: Biomech Model Mechanobiol. 2026 Jun 3;25(3):61. doi: 10.1007/s10237-026-02081-7 (PMC13233878; doi:10.1007/s10237-026-02081-7)
Supplement: Supplementary file 4 — Supplementary file4 (DOCX 907 kb) [file 10237_2026_2081_MOESM4_ESM.docx]

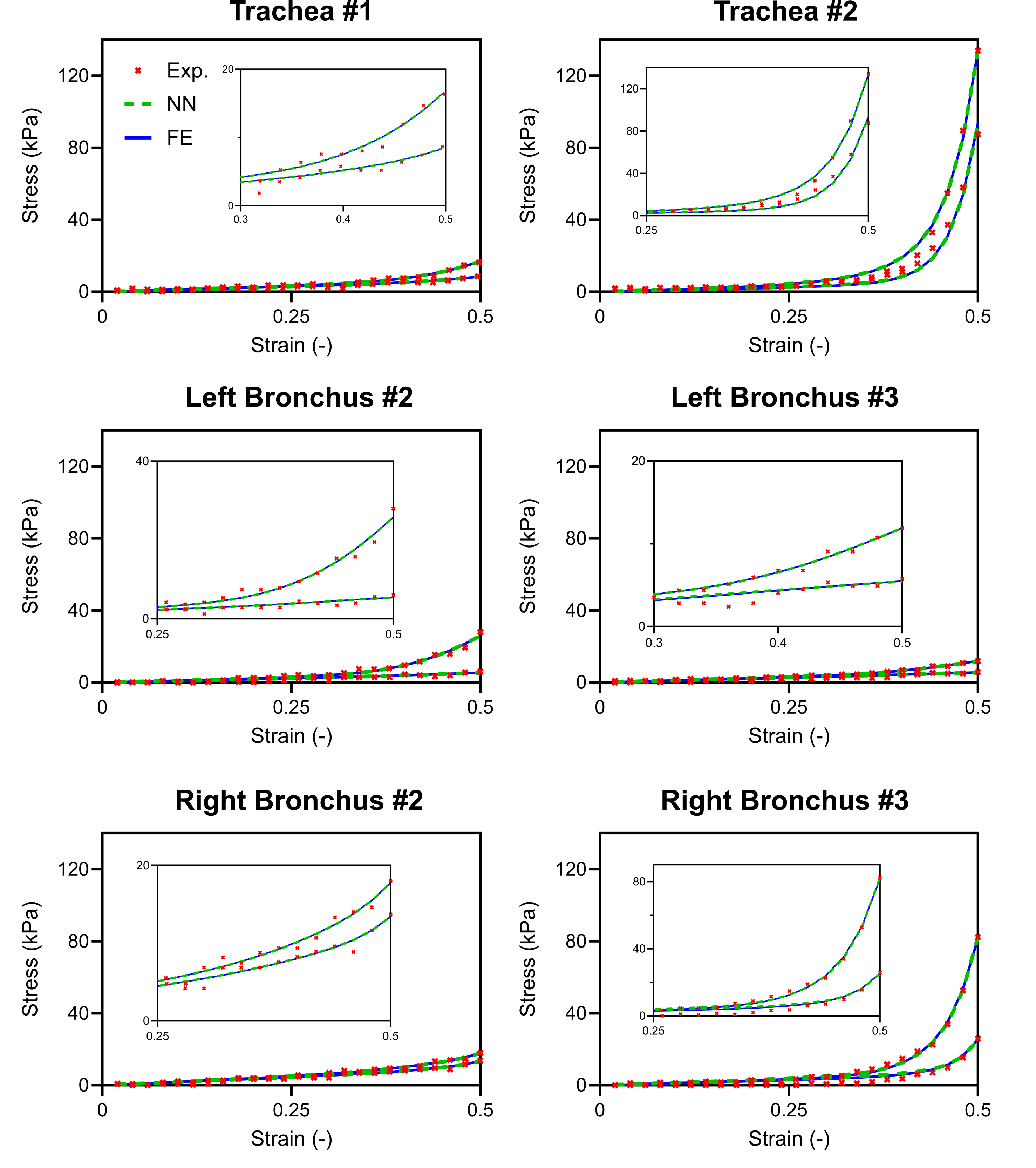
**Results for Reference Samples: Comparison of Experimental, Model, and Neural Network Stress–Strain Curves**

Stress–strain curves of the remaining reference samples among the nine. Sample numbers correspond to those reported in Table 3 of the main manuscript.
